# Gamma-aminobutyric acid modulates buck semen quality and sperm preservation duration in vivo and in vitro

**DOI:** 10.1093/jas/skag294

**Published:** 2026-09-15

**Authors:** Jintao Li, Kangqi Yang, Mengyu Wang, Runyu Yang, Yiqi Zhao, Huaiping Shi

**Affiliations:** Shaanxi Key Laboratory of Molecular Biology for Agriculture, College of Animal Science and Technology, Northwest A&F University, Yangling 712100, China; Shaanxi Key Laboratory of Molecular Biology for Agriculture, College of Animal Science and Technology, Northwest A&F University, Yangling 712100, China; Shaanxi Key Laboratory of Molecular Biology for Agriculture, College of Animal Science and Technology, Northwest A&F University, Yangling 712100, China; Shaanxi Key Laboratory of Molecular Biology for Agriculture, College of Animal Science and Technology, Northwest A&F University, Yangling 712100, China; Shaanxi Key Laboratory of Molecular Biology for Agriculture, College of Animal Science and Technology, Northwest A&F University, Yangling 712100, China; Shaanxi Key Laboratory of Molecular Biology for Agriculture, College of Animal Science and Technology, Northwest A&F University, Yangling 712100, China

**Keywords:** GABA, buck, semen quality, rumen microbiota, in vitro preservation, PLA2G10

## Abstract

Improving sperm motility and preservation duration is crucial for enhancing reproductive efficiency in dairy goats. Given the decline of gamma-aminobutyric acid (GABA) during in vitro storage, this study evaluated its effects and mechanisms on buck semen via diet and extender supplementation. Dietary GABA supplementation significantly increased ruminal total volatile fatty acids (*P *< 0.01) and ammonia nitrogen levels (*P *< 0.05), enriched beneficial bacteria, enhanced serum antioxidant capacity (*P *< 0.05), and improved sperm motility, velocity, acrosome and plasma membrane integrity (*P *< 0.05). Although GABA did not cross the blood–testis barrier, it significantly elevated serum LH and testosterone levels (*P *< 0.01). In the in vitro preservation experiment, adding 1.0 g/L GABA to the extender significantly improved sperm viability, kinematic parameters, antioxidant capacity, acrosome and membrane integrity on days 3 and 5 of storage (*P *< 0.05), while reducing reactive oxygen species (ROS) and malondialdehyde (MDA) levels (*P *< 0.05). Proteomic analysis revealed that GABA upregulated membrane structure-related proteins, and PLA2G10-mediated changes in phospholipase A2 (PLA2) activity were identified as a key mechanism by which GABA modulates sperm membrane structure and motility. Inhibition of PLA2G10 with LY329722 significantly abrogated the protective effects of GABA (*P *< 0.01). GABA not only indirectly improves buck semen quality by modulating the rumen microbiota and hypothalamic–pituitary–testicular axis but also directly enhances in vitro sperm preservation through PLA2G10-mediated membrane remodeling. GABA can serve as a functional feed additive and a semen extender protectant to effectively improve reproductive management in ruminants.

## Teaser

Dietary GABA boosts goat semen quality by reshaping rumen microbes and hormones, while direct addition to semen extender preserves sperm via PLA2G10-driven membrane remodeling—offering a dual strategy for better livestock reproduction

## Introduction

The conception rate of dairy goats after artificial insemination is generally low, mainly due to the poor quality of semen, followed by a rapid decline in sperm motility during liquid storage of semen and an unsatisfactory recovery rate after thawing during cryopreservation (Yuan et al. 2023). Consequently, advancing technology to enhance semen quality and extend in vitro semen preservation duration is of paramount importance for increasing the dissemination and longevity of superior breeding livestock. In the domain of animal husbandry, feed additives are frequently employed to enhance animal production performance. To improve semen quality, commonly utilized feed additives include antioxidants such as vitamin E (Hayanti et al. 2022), vitamin C (Zhang et al. 2022b), organic selenium yeast (Sabzian-Melei et al. 2022), and zinc methionine (Prabakar et al. 2021); energy metabolism regulators such as L-Carnitine (Samiei et al. 2025) and conjugated linoleic acid (CLA) (Karimi et al. 2017); as well as other additives like resveratrol (Guo et al. 2024), lycopene (Zhai and Tang 2020), omega-3 polyunsaturated fatty acids (ω-3 PUFA) (Jing et al. 2024), folic acid (vitamin B9) (Najafipour et al. 2017), and probiotics (lactobacilli) (Mahiddine et al. 2022), among others. These additives significantly contribute to enhancing sperm motility, density, membrane integrity, and antioxidant capacity, while also reducing deformity rates. The metabolic activity of spermatozoa during in vitro preservation is essential for their viability (Ferreira et al. 2018). Prior research has employed antioxidants, energy metabolism regulators, and bioactive plant compounds to modulate semen quality. However, there is a lack of research identifying specific small molecule metabolites that are vital for spermatozoal function. Consequently, this study employed metabolomic sequencing of viable sperm from semen samples subjected to varying storage durations and identified the metabolite gamma-aminobutyric acid (GABA) as being correlated with sperm motility. This finding suggests that GABA has potential as a functional feed additive and semen preservation agent to improve the semen quality of bucks and extend in vitro semen preservation time.

GABA is an endogenous neurotransmitter synthesized via the decarboxylation of glutamate, a reaction catalyzed by the enzyme glutamate decarboxylase (GAD). It is ubiquitously present in the nervous system and functions to inhibit neuronal excitability through its interaction with GABA receptors (Chen et al. 2019). The efficacy of GABA is contingent upon the regulation of the GABAergic system, which constitutes the primary inhibitory signaling mechanism within the central nervous system (CNS) (Mazzoli and Pessione 2016). This system is composed of GABA, GADs, GABA transporters (GATs), and GABA receptors (GABARs) (Perovic et al. 2025; Tao et al. 2025). In the context of animal production, empirical evidence suggests that GABA administration can markedly alleviate stress, enhance feed intake, and improve metabolic processes by modulating neural excitation, thereby augmenting growth performance (Fathi et al. 2023). Dietary supplementation with GABA has been demonstrated to enhance growth performance and optimize rumen fermentation parameters in neonatal calves (Zhang et al. 2025). Furthermore, GABA has been shown to significantly improve the intestinal microbiota in diabetic mice, although its impact on rumen microbiota remains unexplored (Zhang et al. 2022a). In recent research, the distribution of GABA across various segments of the male reproductive system has been examined. The findings indicate that GABA levels are highest in the vas deferens and lowest in testicular tissue, suggesting a potential role for GABA in influencing sperm motility (Erdö et al. 1983). Furthermore, another study demonstrated a significant positive correlation between the proliferation of GABA-producing microorganisms and elevated serum levels of luteinizing hormone (LH) and follicle-stimulating hormone (FSH), implying that GABA may exert an influence on gonadal function (Liang et al. 2021). Investigations into the testes of male mice have revealed that GABA might regulate the activity of testicular interstitial cells by enhancing their testosterone (T) secretion, thereby elevating T levels (Doepner et al. 2005). In conclusion, GABA appears to positively influence the reproductive performance of breeding rams during the breeding season and extend the preservation duration of semen in vitro. We propose that GABA enhances the rumen microbiota of breeding bucks, thereby improving their overall health and reproductive capabilities. Additionally, GABA may serve as a protective agent for in vitro semen preservation, enhancing the efficacy of liquid semen storage. Consequently, this study aims to investigate the effects of dietary GABA supplementation on rumen-related indicators and microbial community, serum antioxidant capacity, semen quality, and sperm antioxidant capacity in breeding bucks. Furthermore, GABA was incorporated into the diluent to assess its impact on semen preservation in vitro.

## Materials and methods

### Animal ethics statement

All experimental procedures were approved by the Institutional Animals Care and Use Committee (IACUC) of Northwest A&F University, China (Approval No. DK2022008).

### Animals and experiment design

This study was conducted in Qianyang goat breeding farm, Qianyang County, Shaanxi Province, China (latitude 34° 34′ 34″ N and longitude 106° 56′ 15″ E). Healthy and sexually vigorous Saanen breeding bucks under unified management were monitored. In order to screen for key metabolites in semen, after three repeated semen collections, six high-quality bucks (1.5 yr old, sperm motility ≥ 80%) were selected, and then semen was collected using the pseudo vaginal method for non-targeted metabolomics analysis.

To verify the effect of key metabolites in semen on bucks, 20 healthy Saanen breeding bucks (1.5 yr old, sperm motility ≥ 80%) were randomly assigned into two groups, with each buck raised in a single pen and 10 replicates in each group. Bucks in each group were received with a basal diet supplemented with 100 mg/kg GABA or not (0 mg/kg). GABA was purchased from Zhejiang Esigma Biotechnology Co., Ltd with a purity of 50%, with the remaining 50% being corn starch as the carrier. The composition and nutritional levels are shown in Table 1. All bucks were kept in a clean and comfortable environment. After 56 d, blood, rumen fluid, and semen were collected for subsequent analysis.

**Table 1:** Composition and nutritional levels of the basal diet (dry matter basis, %).

Item	Content
**Ingredients**	
**Yellow corn**	46.00
**Bran**	20.00
**Soybean meal**	20.00
**Wheat**	10.00
**CaHPO₄**	2.00
**Salt**	1.00
**NaHCO₃**	1.00
**Total**	100.00
**Nutrient levels^1^^,^^2^**	
**NE (MJ/kg)**	6.88
**Crude protein**	10.04
**Neutral detergent fiber**	34.79
**Acid detergent fiber**	25.59
**Ca**	0.55
**P**	0.23

1 Each kilogram of mixture contains: vitamin A, 3000 IU; vitamin D, 320 IU; vitamin E, 18 IU; Fe, 40 mg; Cu, 6 mg; Zn, 35 mg; Mn, 35 mg; I, 0.3 mg; Se, 0.1 mg; Co, 0.1 mg.

2 NE is a calculated value, and others are measured values.

For the semen preservation test at 4°C in vitro, six 1.5-yr-old bucks with sperm motility ≥ 80% were selected. Semen was collected by pseudo vaginal method. Semen was diluted after mixing, and then divided into groups with six replicates in each group. NC group and GABA group were set up, and proteomic sequencing was performed after 3 d of storage. Subsequently, NC group, GABA group, LY329722 group, and GABA+LY329722 group were set for follow-up test. GABA was purchased from Beijing Biotopped Technology Co., Ltd (Beijing, China, purity ≥ 99.0%, Cat No: A1182A). LY329722 was custom-ordered from Shanghai Nafu Biotechnology Co., Ltd.

### Semen dilution and preservation

The semen was serially diluted with the extender at a 1:1 ratio (equal-volume dilution) at 35–37°C, and this step was repeated three times until the final semen-to-extender ratio reached 1:7. After each dilution step, the mixture was allowed to stand for 3 min before the next dilution. The semen diluent was then cooled in a gradient to 4°C for storage (at a rate of 8–10°C/h). Cooling to 4°C was achieved by placing the samples in a 500 mL beaker containing 350 mL of water at 37°C (isothermal with the semen), with the water level slightly higher than that of the semen sample. The beaker was then placed in a 4°C refrigerator, and the cooling rate of 8–10°C/h was obtained by allowing the water to cool naturally from 37 to 4°C, which was monitored by recording the temperature at regular intervals using a thermometer. Non-targeted metabolomics sequencing was performed on samples collected on days 0 and 5. The diluent formula is shown in Table 2. All reagents listed in Table 2 were purchased from Sigma-Aldrich.

**Table 2:** Main components of semen diluent.^1^

Item	Content (g)
**Tris**	3.03
**Citric acid**	1.60
**Glucose**	0.64
**D-Fructose**	0.64
**Trehalose**	0.25
**BSA**	0.10
**Soy lecithin**	0.375
**H_2_O**	100 mL

^1^The diluent needs to be prepared and used now. Weigh the reagent in the table and dissolve it in 100 mL of water. Put it in a magnetic stirrer and stir it at 37°C until it is completely dissolved. Use a 0.45 μm filter to filter it into a sterile container. Add 1 × 10^5^ IU of penicillin and streptomycin, respectively. Mix well and then use it for standby.

### Semen quality analysis

Five microliters of semen were placed on a glass slide. Then, the Miran semen assisted analysis system was used to analyze semen quality-related parameters, including sperm viability, motility, curvilinear velocity (VCL), straight-line velocity (VSL), and average path velocity (VAP). At least five areas were selected, each with a minimum sperm count of 200.

### Rumen fermentation parameters analysis

On day 56, rumen fluid was obtained using a stomach tube and then filtered through four layers of gauze. (1) The pH value was determined using a pH-3B high precision acidimeter. (2) The rumen fluid samples were centrifuged at 12,000 × *g* for 10 min at 4°C. After that, 100 μL of the resulting supernatant was mixed with 100 μL of deionized water and 800 μL of 0.2 mol/L hydrochloric acid. Ammonia nitrogen (NH3-N) levels were identified using the phenol-sodium hypochlorite colorimetric method. (3) Four hundred microliters of 25% metaphosphoric acid were added to 2 mL of supernatant for centrifugation at 10,000 × *g*, 4°C for 10 min. The contents of volatile fatty acids (VFAs) were detected using gas chromatography (Agilent, 7890). (4) Rumen fluid samples were stored at −80°C for 16S rDNA sequencing.

### Sperm plasma membrane integrity and acrosome integrity analysis

The plasma membrane integrity of sperm was assessed using SYBR-14/PI fluorescence staining method. Acrosome integrity was analyzed using FITC-PNA fluorescence staining. The corresponding kits used here were provided by Shanghai Jiemei Gene Pharmaceutical Technology Co., Ltd (Shanghai, China). Three visual fields were randomly selected from semen-stained slides of each goat, and ImageJ was used to calculate the percentage.

### Antioxidant index analysis in serum and sperm

Upon completion of the experiment, 5 mL of blood samples were collected from the jugular vein utilizing vacuum tubes. Serum was subsequently obtained by centrifugation at 3000 × *g* for 10 min. Sperm samples were collected via the pseudo-vaginal method, resuspended in precooled phosphate-buffered saline (PBS), and subjected to two centrifugation washes at 500 × *g* for 5 min each time to ensure the complete removal of seminal plasma. The levels of superoxide dismutase (SOD), catalase (CAT), glutathione peroxidase (GSH-Px), malondialdehyde (MDA), total antioxidant capacity (T-AOC), and reactive oxygen species (ROS) were quantified using the respective assay kits provided by Nanjing Jiancheng Bioengineering Institute, Jiangsu, China.

### Analysis of hormone in serum

The above isolated serum was analyzed for LH, FSH, and T using the corresponding enzyme-linked immunosorbent assay (ELISA) Kit (Fankewei Biotechnology Co., Ltd, Shanghai, China).

### Non-targeted metabolomics analysis

Semen metabolomics was performed by Guangzhou Gidi Bio-Tech Co., Ltd (Guangzhou, China). Briefly, 100 μL of semen was mixed with 400 μL of extraction solution containing isotopic labeled mixture (methanol:acetonitrile = 1:1). Afterwards, the mixture is sonicated in an ice water bath for 10 min. To obtain supernatant, the samples were centrifuged at 13,000 × *g*, 4°C for 15 min. Metabolites were separated using Accucore C-18 column (Thermo Scientific, USA) (column temperature = 25°C; flow rate = 0.30 mL/min; injection volume = 2 μL). A 0.1% formic acid solution was added to each mobile phase to optimize separation and reduce ion inhibition. The following system is conducted during the elution process: 5% B (0–5 min), 90% B (520 min), 90% B (20–30 min), 5% B (30–35 min). Metabolite identification was performed using high resolution mass spectrometry (HRMS) equipped with an Orbitrap mass analyzer. The detection was conducted in electro spray ionization (ESI) positive and negative modes. Before peak recognition, extraction, and alignment, the raw data were converted into mzXML format using ProteoWizard software. The processed data were matched with secondary mass spectrometry database of BiotreeDB (Version 2.1) for accurate annotation of metabolic products. The PCA and *t*-test were performed between the two groups, with the FDR adjusted *P*-value <0.05 and the VIP >1 being considered as significantly different metabolites.

### 16S rDNA sequencing of rumen fluid

16S rDNA sequencing was conducted by Guangzhou Gidi Bio-Tech Co., Ltd (Guangzhou, China). After extracting genomic DNA from rumen fluid samples, PCR amplification of V3-V4 variable region was performed using specific primers with barcode (341F, 5′-CTACGGGNGGCWGCAG-3′; 806R, 5′-GGACTACHVGGGTATCTAAT-3′). Before sequencing on Illumina platform (Illumina, USA), the purified amplification products were connected to sequencing adapters, and a library was constructed. After the raw data were quality-controlled, denoised, and merged, operational taxonomic units (OTU) with 1611 similarity were obtained. Based on the OTU level, α-diversity, including Chao1, Sob, Simpson, and Ace indices, was evaluated with Omicsmart software. Beta diversity was analyzed through calculating the weighted Unifrac distance and visualized by principal co-ordinates analysis (PCoA). Welch’s *t*-test was adopted to compare the bacterial abundance.

### Proteomic sequencing

Washed sperm cells were added to SDT lysis buffer (100 mM NaCl) containing DTT, lysed, and sonicated. After centrifugation, the supernatant was heated, alkylated with iodoacetamide, and precipitated with acetone. Protein concentration was determined using the Bradford assay, and integrity was verified by SDS-PAGE. An aliquot of 20 μg protein was digested with trypsin in 100 mM TEAB (pH 8.5) at 37 °C for 4 h. The resulting peptides were desalted on a C18 column and lyophilized. The dried peptides were dissolved in 0.1% formic acid and analyzed on a Vanquish Neo UHPLC system coupled to an Orbitrap Astral mass spectrometer.

### Analysis of sperm phospholipase A2 (PLA2) activity

PLA2 activity assay kit (Beijing Box Biotechnology Co., Ltd) was used to detect the PLA2 Activity in sperm.

### Data analysis

The data were presented as the mean ± standard deviation (SD) and were analyzed, with graphical representations generated, using GraphPad Prism 8 software. Statistical differences between the control and GABA-treated groups were assessed using an independent samples *t*-test. Two-way ANOVA was used to verify the statistical difference in the in vitro preservation test, and multiple comparisons were made. A *P*-value of less than 0.05 was considered to indicate statistical significance.

## Results

### Depletion of GABA during sperm storage highlights its critical role in supporting sperm survival

In this study, a semen preservation dilution solution was employed for the low-temperature storage of sperm (Figure 1C). Our findings indicated that sperm viability and motility exceeded 50% by the 5th day (Figure 1A and B). To elucidate the key metabolites involved in sperm survival, non-targeted metabolomics sequencing was performed on viable sperm from days 0 and 5 (Figure 1C). This analysis revealed a total of 62 annotated metabolites, with distinct metabolomic profiles discerned between the two groups based on PCA analysis (Figure 1D). Notably, a reduced abundance of several metabolites, including GABA and proline betaine, was observed in sperm on day 5 compared to day 0 (Figure 1E). Further KEGG pathway analysis of these altered metabolites identified several functional categories, with the top five enriched pathways being morphine addiction, aminoacyl-tRNA biosynthesis, ABC transporters, arginine and proline metabolism, and GABAergic synapse (Figure 1F). Therefore, it is postulated that GABA, due to its role as a metabolite advantageous for sperm viability, may function as an effective dietary supplement to improve the reproductive outcomes in male goats.

**Figure 1:**
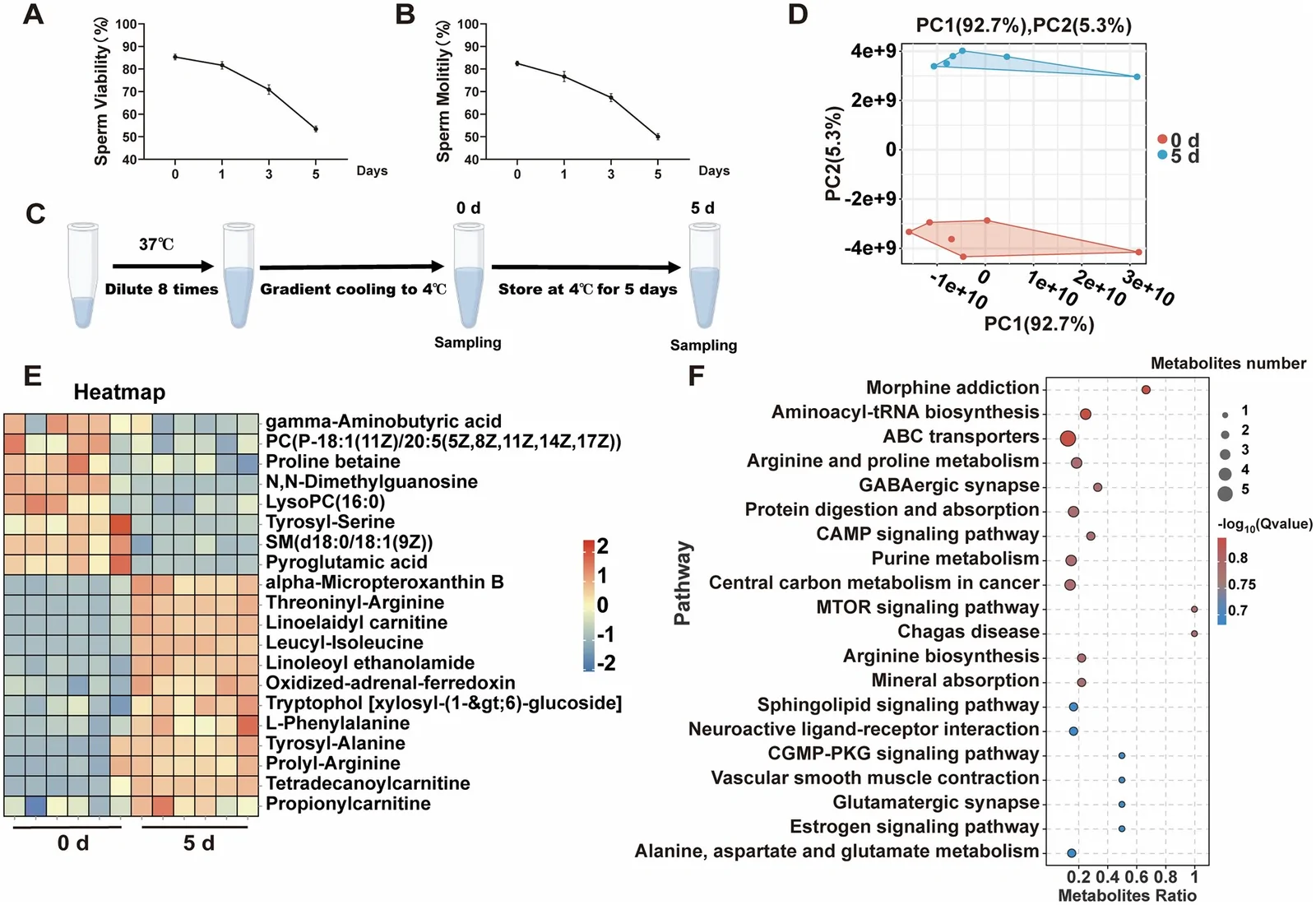
Screening of metabolites related to sperm survival. (A, B) Sperm viability and motility analysis through CASA; (C) Semen dilution and preservation; (D, E) PCA plot and heatmap from non-targeted metabolomics of semen on days 0 and 5; (F) KEGG pathway enrichment analysis (*n *= 6).

### GABA improves rumen fermentation parameters and modulates rumen microbiota composition in bucks

In accordance with the manufacturer’s instructions, the bucks were provided with a basal diet supplemented with 100 mg/kg of GABA. Following a 56-d duration, ruminal fermentation parameters were evaluated. The results demonstrated that GABA supplementation did not exert a significant impact on the ruminal pH value (Figure 2A). Nonetheless, it significantly elevated the concentration of ruminal NH_3_-N (*P *< 0.05; Figure 2B). Additionally, there were significant increases in the levels of isobutyrate, butyrate, isovalerate, and total VFAs in the rumen of bucks receiving GABA supplementation, compared to those in the control group (*P *< 0.05; Figure 2C and D).

**Figure 2:**
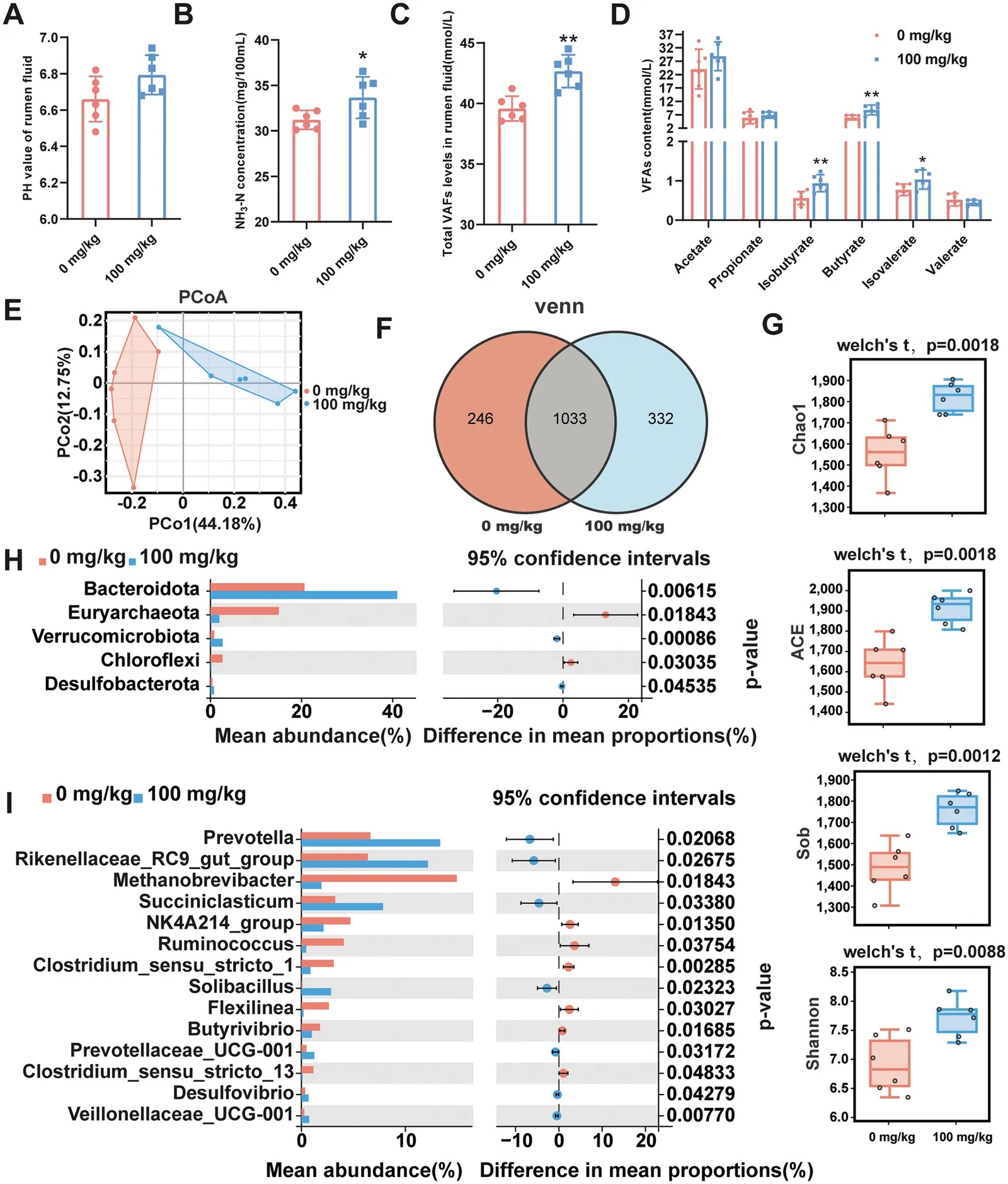
Effect of GABA supplementation on rumen fermentation parameters in bucks. (A) pH value of rumen fluid; (B) NH_3_-N concentration; (C) Total VFAs levels in rumen fluid; (D) The contents of acetate, propionate, isobutyrate, butyrate, isovalerate, and valerate in rumen fluid; (E) PCoA plot; (F) Venn plot; (G) α-diversity analysis; (H) Rumen microbiota composition at phylum level; (I) Rumen microbiota composition at genus level (*n* = 6). Data were expressed as the mean ± SD (*n *= 6). *P < 0.05, **P < 0.01.

Subsequently, 16S rDNA sequencing was performed to elucidate the alterations in rumen microbiota induced by GABA administration. As illustrated in Figure 2F, the control and GABA-fed bucks exhibited 1279 and 1365 OTUs, respectively, with 1033 OTUs shared between the two groups. GABA treatment resulted in a significant enhancement of α-diversity within the rumen microbiota, as evidenced by increased Chao1, ACE, Sob, and Shannon indices (*P *< 0.05; Figure 2G). Furthermore, the PCoA plot demonstrated a distinct separation between the two groups (Figure 2E). To further explore the impact of GABA on the composition of rumen microbiota in bucks, we conducted an analysis of microbiota variations at both the phylum and genus levels. At the phylum level, GABA administration significantly elevated the relative abundance of *Bacteroidota*, *Verrucomicrobiota*, and *Desulfobacterota*, while reducing the proportions of *Euryarchaeota* and *Chloroflexi* (*P *< 0.05; Figure 2H). At the genus level, significant differences were observed in 14 bacterial genera, with seven genera exhibiting an increase and seven genera showing a decrease in the GABA-treated bucks (*P *< 0.05; Figure 3I, Figure S1A). Functional prediction of the microbial community showed that GABA significantly enhanced the functions of carbohydrate metabolism, membrane transport, replication and repair, signal transduction, and xenobiotics biodegradation and metabolism (*P *< 0.05; Figure S1B). These results indicate that GABA improves ruminal energy supply and nutrient absorption capacity, improves the rumen microbial community, and enhances rumen barrier function.

**Figure 3:**
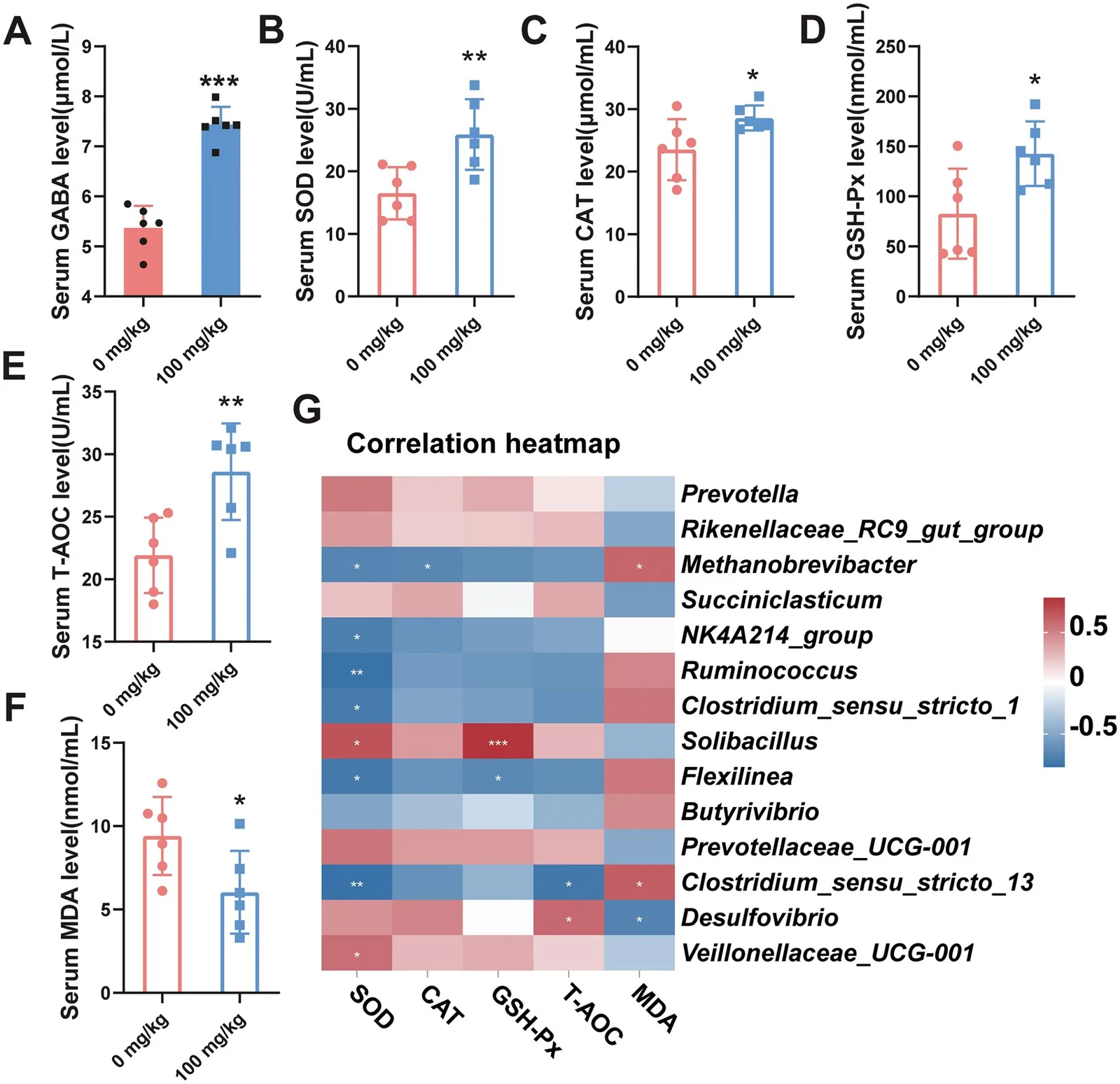
Effect of GABA supplementation on serum GABA and antioxidant indexes in bucks. (A) GABA level in serum; (B–D) SOD, CAT, and GSH-Px activities in serum; (E) T-AOC in serum; (F) MDA level in serum; (G) Correlation heatmap between rumen flora and serum antioxidant indexes. Data were expressed as the mean ± SD (*n* = 6). *P < 0.05, **P < 0.01, and ***P < 0.001.

### GABA enhanced serum antioxidant capacity in bucks

Dietary supplementation with GABA resulted in a significant elevation of serum GABA levels (*P *< 0.001; Figure 3A). Furthermore, T-AOC and the activities of serum antioxidant enzymes, including SOD, CAT, and GSH-Px, were markedly higher in GABA-supplemented bucks compared to the control group (*P *< 0.05; Figure 3B–E). Concurrently, GABA supplementation led to a significant reduction in serum MDA levels (*P *< 0.05; Figure 3F). Additionally, Spearman’s correlation analysis identified 16 significant correlations, comprising six positive and ten negative relationships between rumen microbiota and serum antioxidant indices. Notably, serum SOD levels were negatively correlated with the relative abundances of *Methanobrevibacter*, *NK4A214_group*, *Ruminococcus*, *Clostridium_sensu_stricto_1*, *Flexilinea*, and *Clostridium_sensu_stricto_13*. In contrast, the relative abundance of *Solibacillus* in the rumen was positively associated with serum SOD and GSH-Px levels (Figure 3G).

### GABA supplementation improved semen quality in bucks

To investigate the potential impact of GABA on semen quality via the blood–testis barrier (BTB), we measured the GABA content in sperm from bucks. As depicted in Figure 4A, no significant difference was observed in sperm GABA content between two experimental groups, suggesting that GABA does not traverse the BTB (*P *> 0.05). Nevertheless, GABA administration resulted in a significant enhancement of sperm viability, motility, VSL, and VAP in bucks (*P *< 0.05; Figure 4B). Additionally, GABA markedly improved acrosome integrity and membrane integrity in sperm (*P *< 0.05; Figure 4C). Subsequently, we assessed the antioxidant activities of sperm in response to GABA supplementation. No significant effect of GABA on ROS levels in sperm was observed (*P *> 0.05; Figure 4D), while it significantly enhanced the activities of SOD and GSH-Px, as well as the T-AOC (*P *< 0.05; Figure 4E, G, and H), with a trend toward increased CAT activity (0.05 < *P *< 0.1; Figure 4F). Furthermore, GABA significantly reduced MDA level in sperm (*P *< 0.05; Figure 4I). Spearman correlation analysis was used to explore the relationship between rumen microbiota and sperm quality parameters. Positive correlations were identified between the genera *Prevotella*, *Rikenellaceae_RC9_gut_group*, *Solibacillus*, *Prevotellaceae_UCG-001*, *Veillonellaceae_UCG-001* and parameters such as VAP, membrane integrity, and the activities of sperm SOD and GSH-Px. Conversely, the relative abundances of *Methanobrevibacter*, *NK4A214_group*, and *Clostridium_sensu_stricto_1* exhibited negative correlations with sperm viability, sperm motility, VSL, VAP, membrane integrity, and the levels of sperm SOD and GSH-Px (Figure 4J). These results suggest that the improvement of sperm quality caused by dietary GABA supplementation may be caused by affecting rumen microorganisms.

**Figure 4:**
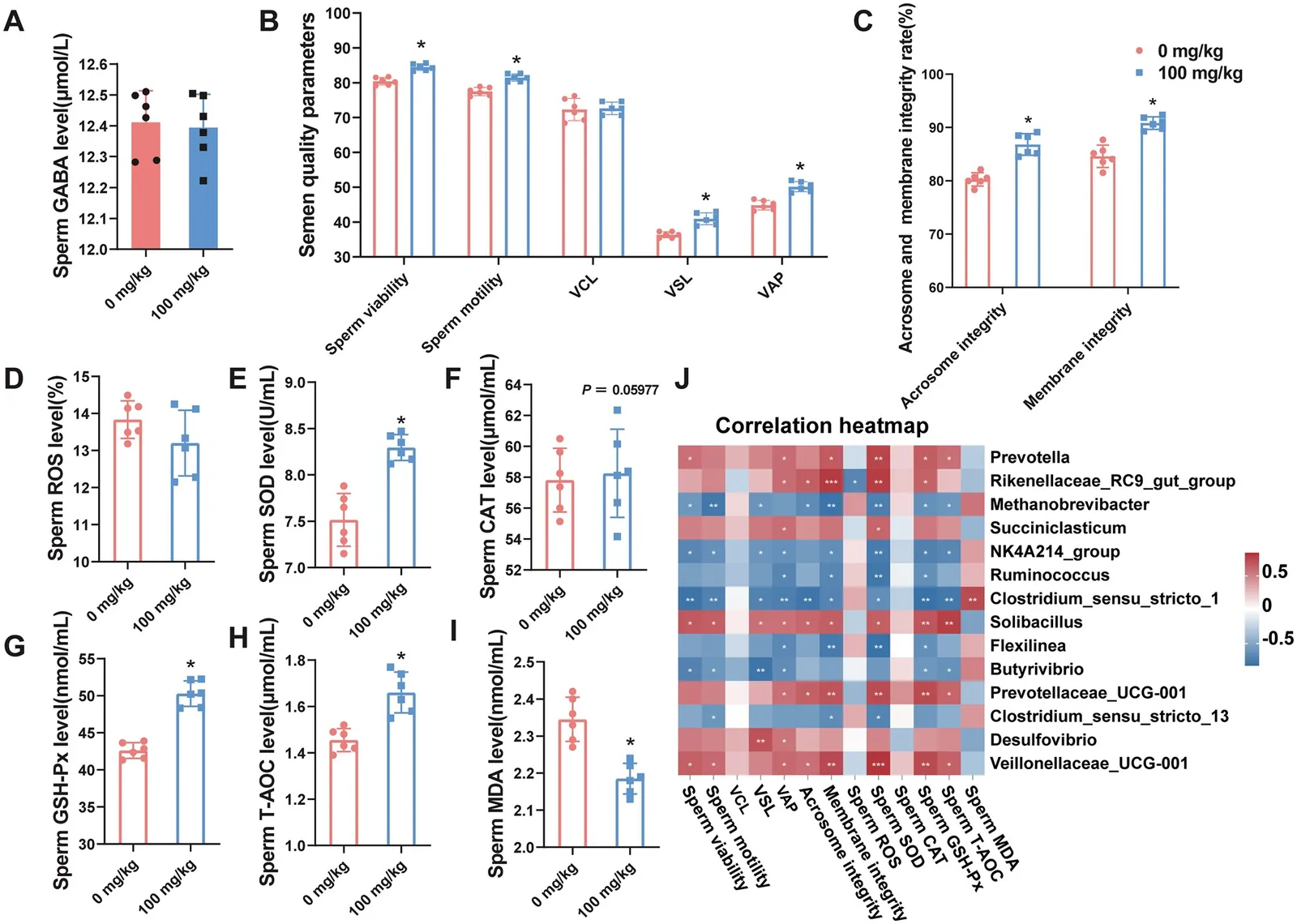
Effect of GABA supplementation on semen quality and antioxidant indexes in bucks. (A) GABA level in sperm; (B) Semen quality analysis, including sperm viability, sperm motility, VCL, VSL and VAP; (C) Sperm acrosome integrity and membrane integrity analysis; (D) Sperm ROS level; (E–G) SOD, CAT, and GSH-Px activities in sperm; (H) T-AOC level in sperm; (I) MDA level in sperm; (J) Correlation heatmap between rumen flora and semen indexes; Data were expressed as the mean ± SD (*n* = 6). *P < 0.05, **P < 0.01, and ***P < 0.001.

### GABA supplementation increases serum LH and T levels

The aforementioned data indicated that GABA did not have a direct effect on the GABA concentration in sperm. However, sperm quality was significantly enhanced following a diet supplemented with GABA. Consequently, to investigate whether GABA could enhance semen quality via the hypothalamic–pituitary–testicular (HPT) axis, serum hormone levels were measured using ELISA. The results revealed a significant increase in serum LH and T levels (*P *< 0.05; Figure 5A and C), whereas serum FSH level remained unchanged (*P *> 0.05; Figure 5B).

**Figure 5:**
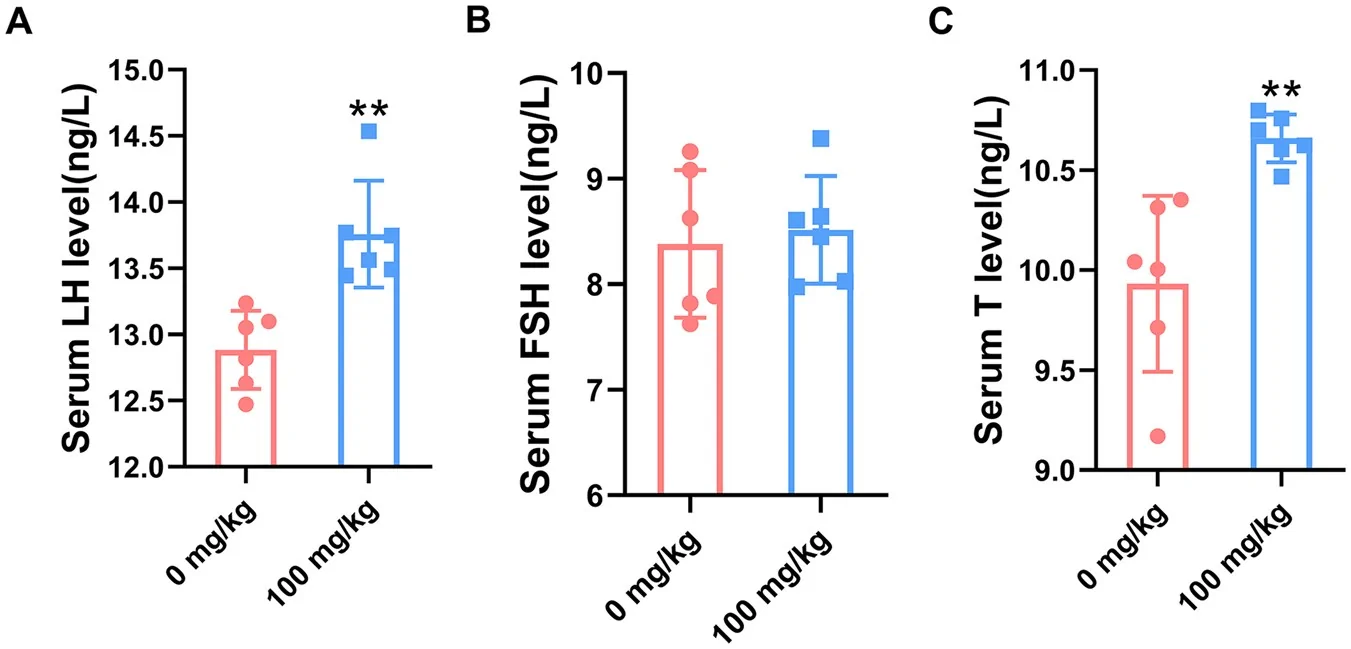
Effects of GABA supplementation on serum hormone levels in bucks. (A) Serum LH level; (B) Serum FSH level; (C) Serum T level. Data were expressed as the mean ± SD (*n* = 6). **P < 0.01.

### Adding GABA to the diluent to improve the effect of semen preservation in vitro

To investigate the direct impact of GABA on sperm, GABA was incorporated into the semen diluent for in vitro cryopreservation. The findings indicated that a GABA concentration of 1.0 g/L resulted in the highest sperm viability and motility, whereas higher concentrations exhibited an inhibitory effect (*P *< 0.05; Figure 6A and B). Furthermore, the sperm’s VSL was significantly greater than that of the control group on the third and fifth days, and the VAP was notably higher on the fifth day (*P *< 0.05; Figure 6C and D). T-AOC was significantly elevated on the third and fifth days of preservation (*P *< 0.05; Figure 6E), while SOD and CAT activities were significantly enhanced at all three time points (*P *< 0.05; Figure 5F and H). GSH-PX activity showed a significant increase on the third day (*P *< 0.05; Figure 6G), and MDA levels were significantly reduced on the third and fifth days of preservation (*P *< 0.05; Figure 5I). ROS levels were significantly decreased at all three time points (*P *< 0.05; Figure 6J). Additionally, the integrity of the acrosome and membrane of the sperm was significantly improved on the third and fifth days (*P *< 0.05; Figure 6K and L). In conclusion, GABA exerts a direct effect on sperm, enhancing the efficacy of low-temperature preservation of dairy goat semen in vitro.

**Figure 6:**
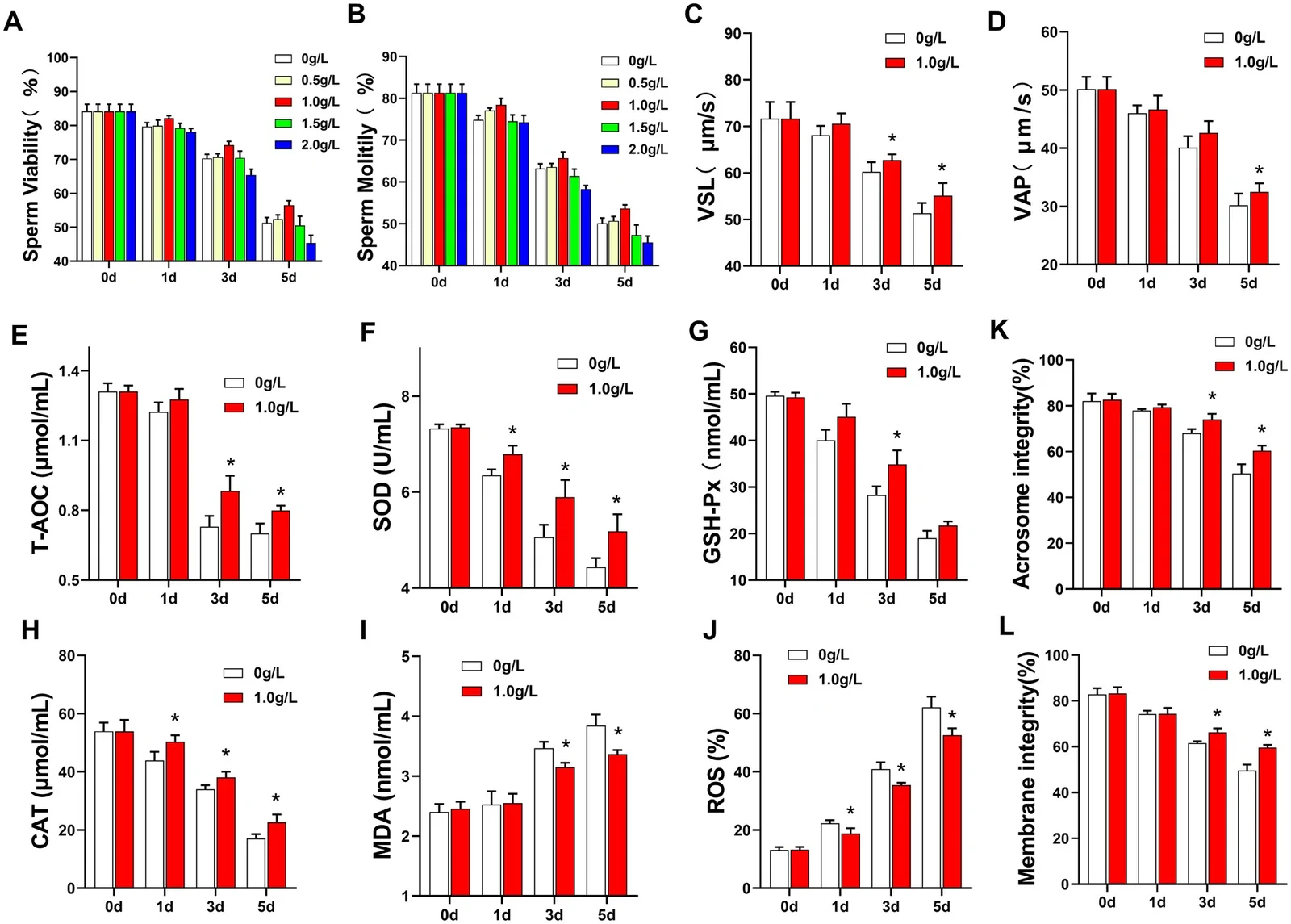
GABA added to the diluent is helpful for semen preservation in vitro. (A, B) Sperm viability and motility (*n* = 6); (C, D) Sperm VSL and VAP (*n* = 6); (E–J) Sperm antioxidant index (*n* = 3); (K, L) Sperm acrosome and membrane integrity (*n* = 3); Data were expressed as the mean ± SD. *P < 0.05.

### Effect of GABA on sperm proteome

In order to study how GABA can improve the effect of semen preservation in vitro, we performed proteomic sequencing. PCA showed that the NC group and GABA group were significantly separated (Figure 7A). Differential protein volcano showed that 27 proteins were up-regulated and 29 proteins were down regulated in GABA group (Figure 7B). The cluster heat map showed 70 proteins, including nine GABA group specific proteins and five NC group specific proteins (Figure 7C). Go analysis showed that the differential proteins were significantly enriched in regulation of apoptotic process, lipid metabolic process, regulation of microtubule polymerization or depolymerization, indicating that differential proteins can improve sperm viability and motility through sperm apoptosis, lipid metabolism, and tubulin mediated changes in sperm kinetic parameters. The cellular components are enriched in cis Golgi network and rough endoplasmic reticulum membrane, indicating that GABA promoted sperm mRNA synthesis of proteins, which also confirmed the presence of specific proteins in GABA group, while the molecular functions are enriched in cis Golgi network and rough endoplasmic reticulum membrane phosphatidylinositol-4,5-bisphosphate 4-phosphatase activity, phospholipase A2 (PLA2) activity, and nucleoside transmembrane transporter activity, indicating that GABA may play an important role in the changes of sperm membrane structure (Figure 7C and D). KEGG analysis enriched in biosynthesis of cofactors, linoleic acid metabolism, alpha-linolenic acid metabolism, pyrimidine metabolism and hippo signaling pathway, indicating that GABA plays an important role in sperm membrane structural changes and apoptosis (Figure 7E). Subcellular localization analysis showed that the differential proteins were mainly nucleus protein, cytoplast protein, extracell protein and plasma membrane protein (Figure 7F). In conclusion, GABA plays a role in sperm apoptosis, lipid metabolism, protein synthesis, and membrane structure changes.

**Figure 7:**
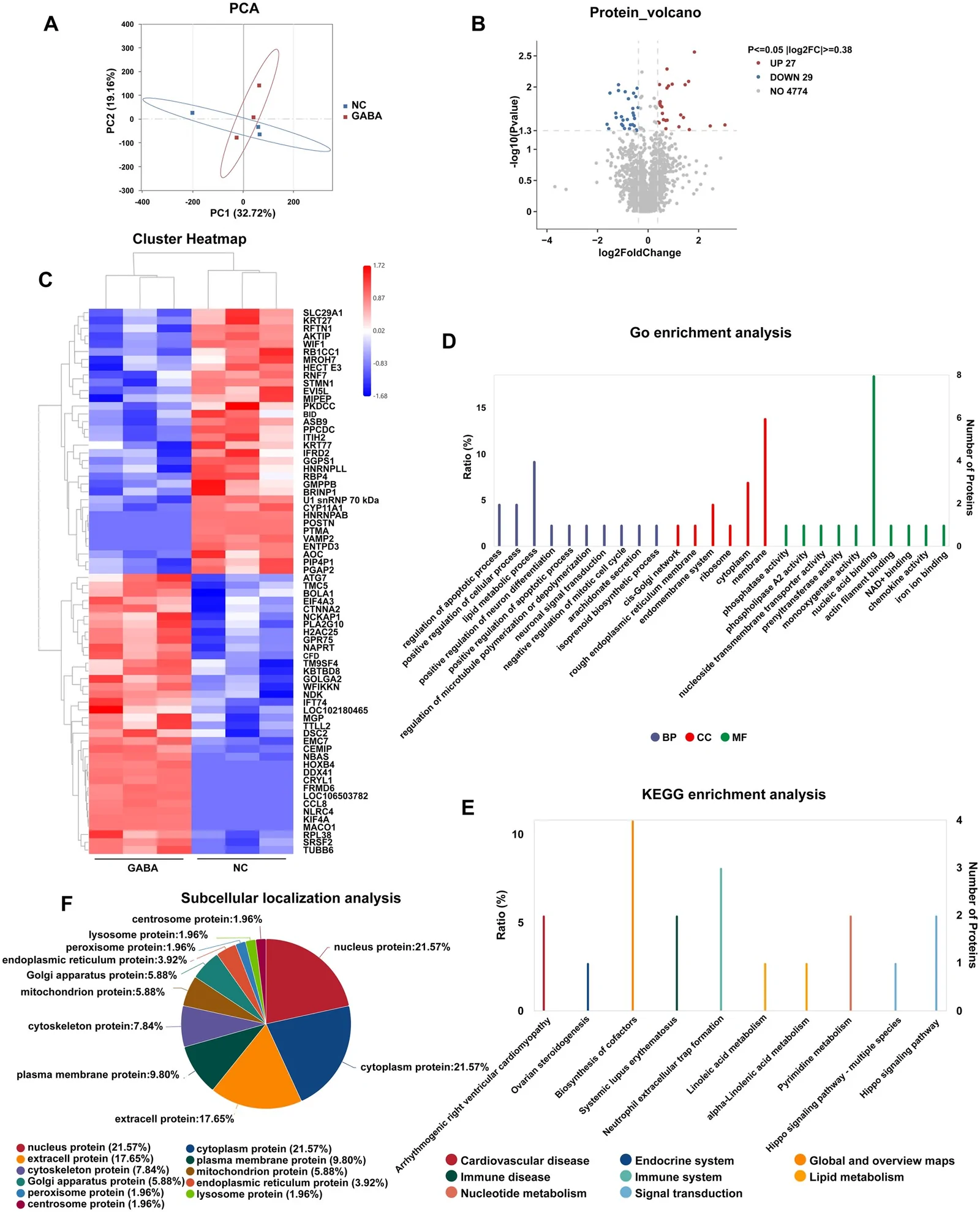
Effect of GABA in semen dilution on sperm proteomics. (A) PCA plot; (B) Differential protein volcano (GABA vs NC); (C) Differential protein cluster heatmap; (D) Go enrichment analysis; (E) KEGG enrichment analysis; (F) Subcellular localization of differential proteins.

### GABA/PLA2G10 regulates sperm motility via membrane structure

Sperm membrane structure plays a critical role in sperm motility. Gene set enrichment analysis (GSEA) revealed that the expression of cell membrane-related proteins was upregulated in the GABA group, whereas it was downregulated in the NC group (Figure 8A). Based on proteomic analysis, PLA2G10 was identified as a key protein, and its inhibitor LY329722 was used to treat sperm. At a concentration of 0.5 μM, LY329722 significantly reduced sperm motility (Figure S2A); therefore, this concentration was selected for subsequent experiments. Further analyses showed that LY329722 treatment significantly decreased sperm viability, motility, acrosome integrity, and plasma membrane integrity, and these effects were markedly alleviated by co-treatment with GABA (Figure 8B–E). Moreover, LY329722 significantly reduced the activities of T-AOC, CAT, GSH-Px, and SOD in sperm, and the reduction in these activities was significantly restored by GABA co-treatment (Figure S2B–E). Meanwhile, LY329722 treatment led to a significant increase in MDA content in sperm, which was significantly decreased upon co-treatment with GABA (Figure S2F). These changes in sperm quality parameters were consistent with the alterations in PLA2 activity, suggesting that GABA may affect sperm function through PLA2G10-mediated changes in PLA2 activity (Figure 8F). Collectively, our results indicate that PLA2G10-mediated changes in PLA2 activity may influence sperm motility by modifying membrane structure.

**Figure 8:**
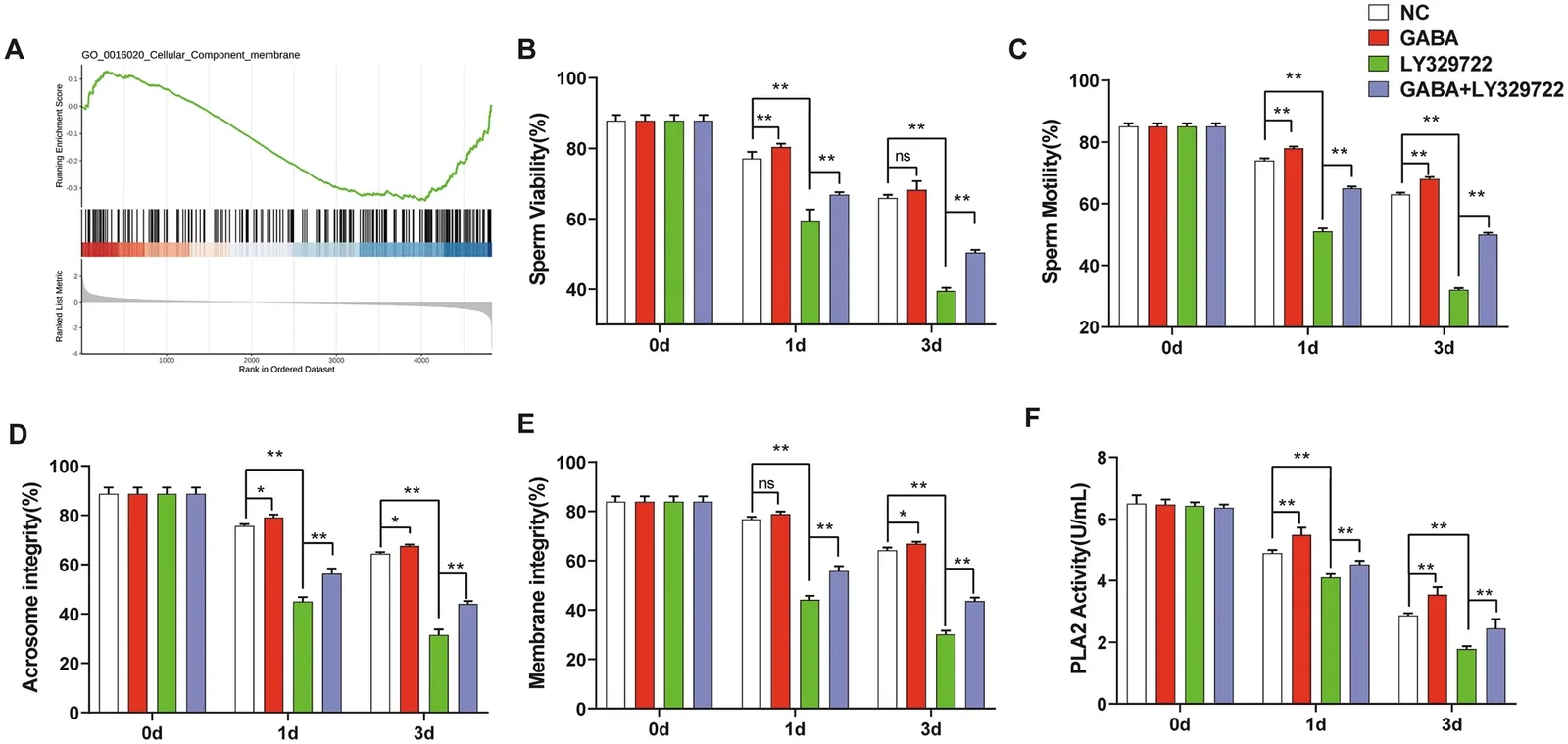
GABA improves sperm motility by affecting sperm membrane structure through PLA2G10. (A) GSEA ES plot (GABA vs NC); (B, C) Sperm viability and motility; (D, E) Sperm acrosome and membrane integrity; (F) PLA2 activity. Data were expressed as the mean ± SD (*n* = 6). *P < 0.05 and **P < 0.01.

## Discussion

This study demonstrated a significant decrease in GABA levels after 5 d of sperm preservation, indicating a potential role for GABA in sperm function. Dietary supplementation with GABA has been shown to improve parameters related to the rumen, enhance bacterial diversity, increase serum antioxidant capacity, and significantly enhance semen quality and sperm antioxidant capacity. It is important to note that GABA is unable to traverse the BTB. Consequently, we supplemented GABA during in vitro preservation at 4°C and observed a marked improvement in overall semen quality. Our findings suggest that GABA regulates a novel mechanism in sperm involving membrane structure remodeling mediated by the phospholipase signaling pathway, rather than relying solely on the classical antioxidant protection pathway.

The physiological mechanisms underlying spermatogenesis and sperm survival are highly intricate, with numerous metabolites playing crucial roles in both spermatogenesis and in vitro preservation. Research indicates that a substantial reduction in leptin levels within the testes can result in impaired sperm production, decreased sperm density, and a markedly reduced offspring survival rate (Argaw-Denboba et al. 2024). Studies have shown that glycolytic metabolite glyceraldehyde-3-phosphate (GA3P) is a vital substrate for bee sperm survival and energy production (Paynter et al. 2017). Furthermore, studies have demonstrated that bees exhibit significant variations in metabolites during different stages of sperm storage, including those involved in glycerophospholipid metabolism, amino acid biosynthesis, and the mTOR signaling pathways (Liu et al. 2020). In our research, we similarly identified metabolic pathways enriched with differential metabolites, such as GABAergic synapses, ATP-binding cassette (ABC) transporters, amino acid metabolism and biosynthesis, aminoacyl-tRNA biosynthesis, and the mTOR signaling pathway, aligning with findings from previous studies. GABA plays a crucial role in GABAergic synapses and is integral to the glutamate/GABA-glutamine cycle, which can enhance glycolysis and glucose utilization (Mamelak 2024). Moreover, glycolysis and glucose utilization are considered primary mechanisms for generating energy in sperm cells (Alves et al. 2025). Consequently, GABA was selected for further experimentation in this study.

The rumen serves as the central organ within the digestive system of ruminants, with its pH value being a critical determinant of feed intake (Bhattacharya and Warner 1967). Carbohydrate fermentation in the rumen results in the production of substantial quantities of volatile fatty acids, which are integral to ruminant metabolism. The primary fatty acid products of this fermentation process are acetic acid, propionic acid, and butyric acid (Wang et al. 2021). Research indicates that the administration of varying doses of GABA influences rumen pH in pre-weaning calves, with a trend of increased pH observed. At low doses, NH_3_-N, acetate, propionate, valerate, butyrate, isovalerate, isobutyrate, and total VFAs decrease, while at high doses, these parameters increase, consistent with the findings of this study (Zhang et al. 2025). Another study showed that the addition of GABA in rumen fermentation in vitro could significantly improve the pH value, NH3-N level, propionate, butyrate, acetate, and total volatile fatty acids and showed a significant upward trend over time (Mamuad et al. 2020). The aforementioned findings align with our experimental study, indicating that GABA plays a crucial role in rumen digestion and metabolism. It significantly enhances the concentration of rumen NH_3_-N and VFAs, thereby promoting the utilization of rumen protein and the supply of energy.

The rumen microbiota is a crucial determinant of cellulose digestion by ruminants, such as cows, sheep, and deer, enabling these animals to utilize it as a primary energy source (Liang et al. 2024). Microorganisms within the rumen are responsible for executing all digestive processes, thereby supplying energy (Bhujbal et al. 2022), protein (Gharechahi et al. 2023), and vitamins (Jiang et al. 2022) to the host. Among these microorganisms, *Bacteroidota* constitutes one of the most essential and abundant bacterial phyla, proficient in the degradation of complex plant polysaccharides (Giovannercole et al. 2025). Empirical evidence suggests that as calves mature and their intake of plant-based feed increases, there is a corresponding rise in the relative abundance of *Bacteroidota*, underscoring its significant role in the digestion of plant-derived nutrients (Tan et al. 2021). Furthermore, studies have demonstrated that during in vitro fermentation, the addition of guanidinoacetic acid (GAA) markedly enhances the relative abundances of *Bacteroidota*, *Prevotella*, and *Prevotellaceae_UCG-001* (Dong et al. 2023). Another investigation revealed that supplementing the diet with GAA significantly elevated the relative abundance of *Bacteroidota* in the rumen of rapidly growing lambs after a 60-d feeding period (Li et al. 2023). In this study, post-administration of GABA, there was a significant increase in the relative abundance of *Bacteroidota*, as well as *Prevotella*, *Rikenellaceae_RC9_gut_group*, *Suciniclasticum*, and *Prevotellaceae_UCG-001*, aligning with findings from previous research. These results suggest that GABA supplementation may enhance the feed conversion efficiency in bucks by modulating the rumen microbiota.

GABA enhances the antioxidant capacity of the body by modulating CNS function, thereby promoting the synthesis and activity of antioxidant enzymes. Additionally, the active amino groups within its molecular structure can directly engage in free radical scavenging reactions, thereby mitigating oxidative stress-induced cellular damage (Mamelak 2024). Empirical evidence suggests that dietary supplementation with GABA can decrease MDA content in silkworm larvae and elevate both the levels and mRNA expression of GSH-Px, CAT, and SOD (Tu et al. 2022). Another investigation demonstrated that pre-transportation GABA supplementation in adult pigs significantly increased serum GSH-Px concentrations while reducing MDA levels (Bi et al. 2020). In the present study, dietary inclusion of GABA resulted in a significant enhancement of antioxidant enzyme content and total antioxidant capacity, concomitant with a reduction in MDA level. Notably, no strong correlation was observed between serum antioxidant indicators and differential microbial genera. In conclusion, GABA supplementation may directly augment the antioxidant capacity of bucks through CNS regulation.

Microorganisms have a significant impact on the reproductive performance of animals. Research has demonstrated that the reproductive capabilities of germ-free mice are diminished (Pronovost et al. 2023; Lopez-Tello et al. 2024). Furthermore, antibiotic-induced dysbiosis of the gut microbiota has been associated with a reduction in testicular weight, loss of germ cells, a decreased number of mature sperm, and a lower survival rate of offspring. Notably, reproductive performance is restored as the microbiota recovers (Argaw-Denboba et al. 2024). Additional investigations have revealed that the microbiota plays a crucial role in extending the reproductive lifespan of mice by safeguarding the ovarian reserve. Furthermore, supplementation with short-chain fatty acids (SCFAs) has been demonstrated to alleviate ovarian dysfunction in germ-free mice (Munyoki et al. 2025). In this context, specific microbial genera exhibit a strong correlation with semen quality parameters. For instance, *Solibacillus* and *Prevotellaceae_UCG_001* show significant positive correlations with most semen quality parameters. Research indicates that *Solibacillus* can produce a bioemulsifier (BE-AM1), which is involved in biofilm formation and cell surface regulation (Markande and Nerurkar 2016). Another study found that the key genus *Prevotellaceae_UCG_001* is positively correlated with total sperm motility and progressive sperm motility, respectively (Pan et al. 2024). Additionally, certain SCFAs were significantly elevated. This suggests that GABA may act as a regulator of rumen microbial communities, potentially enhancing semen quality parameters through the rumen–testis axis.

Hypothalamic gonadotropin-releasing hormone (GnRH) plays a pivotal role in regulating the HPT axis. Specifically, GnRH stimulates the pituitary gland to secrete LH, which subsequently prompts the testes to produce testosterone. Testosterone exerts negative feedback on both the hypothalamus and pituitary gland, thereby inhibiting the secretion of GnRH and LH, respectively (Aleksic et al. 2022). GABA has been shown to stimulate the pituitary gland to release LH and FSH. LH facilitates the proliferation and hormone secretion of testicular interstitial cells, while FSH promotes the production of regulatory molecules and nutrients by Sertoli cells, both of which are essential for spermatogenesis (Lamberts et al. 1983; González-Morán 2020; Ilgin 2020). Furthermore, research indicates that GABA can modulate ovarian health via the hypothalamic–pituitary–gonadal (HPG) axis, with a significant positive correlation observed between GABA levels and both LH and the LH/FSH ratio (Liang et al. 2021; Cheng et al. 2025). In this study, the administration of GABA resulted in a significant increase in serum LH and T levels, alongside notable enhancements in parameters associated with semen quality. These findings suggest that GABA may enhance buck semen quality via modulation of the HPT axis.

As a critical signaling molecule, GABA plays multiple roles in regulating sperm physiological functions (Kurata et al. 2019). Feeding test confirmed that GABA could not pass through the BTB. Although we did not measure GABA levels in testicular tissue or epididymal fluid due to the difficulty of collecting these samples from breeding-season bucks without compromising reproductive performance, we believe this inference is still valid. As a small-molecule metabolite, if GABA could cross the BTB via passive diffusion or transporter-mediated penetration, its levels in sperm would be expected to show an increasing trend. However, the sperm GABA content in the GABA-supplemented group exhibited no such trend at all compared with the control group, strongly supporting our conclusion. Therefore, GABA was directly added to semen diluent for 4°C preservation in vitro. It was found that 1.0 g/L GABA concentration could maintain sperm motility and motility to the greatest extent, while higher concentration showed an inhibitory effect. A dose-dependent improvement was also observed when MitoQ was added to frozen goat semen (Farshad and Wehrend 2025). Moreover, proteomic results revealed nine specifically expressed proteins in the GABA group, indicating that mRNA translation and protein synthesis were initiated; excessive protein synthesis may, however, lead to excessive energy consumption by spermatozoa. These results suggest that the effect of GABA on sperm follows a typical hormetic pattern: an appropriate concentration exerts protective effects by activating GABA receptor-mediated signaling pathways, whereas excessive concentrations may cause overactivation and induce unfavorable physiological responses. Notably, after GABA treatment, both acrosome and plasma membrane integrity were significantly improved on days 3 and 5. The sperm membrane, composed of phospholipids, plays a vital role in maintaining cellular structural integrity and function, and its characteristics directly affect sperm fertility and cryopreservation outcomes. Consistently, proteomic analysis also showed significant enrichment of pathways related to membrane structural changes, including lipid metabolism and linoleic acid metabolism. Subcellular localization analysis further revealed that the differentially expressed proteins were mainly localized to membrane proteins. Taken together, these results indicate that GABA may act directly on sperm membrane structure by regulating membrane lipid metabolism, thereby maintaining functional integrity during low-temperature preservation in vitro.

GSEA analysis showed that membrane-associated proteins were upregulated in the GABA group and downregulated in the NC group, further highlighting the central role of membrane structural changes in GABA-mediated regulation of sperm motility. Based on the proteomic results, we selected PLA2G10 and treated sperm with its inhibitor, LY329722. It was found that 0.5 µM LY329722 significantly inhibited sperm motility; moreover, LY329722 treatment markedly reduced sperm viability, motility, acrosome integrity, and plasma membrane integrity, whereas co-treatment with GABA exerted a significant alleviating effect. These changes in sperm quality parameters were consistent with changes in PLA2 activity. PLA2G10 is highly expressed in the mouse sperm acrosome, is secreted during capacitation, and can amplify the acrosome reaction and its own secretion via an autocrine loop, while also improving the fertilization rate (Escoffier et al. 2011). Previous studies have shown that GABA activates PLA2 in sperm to participate in acrosomal exocytosis, and the MEK-ERK1/2 pathway is involved in regulating PLA2 activation triggered by GABA, progesterone, or the zona pellucida (Chen et al. 2005). In sperm, PLA2 not only participates in the acrosome reaction but also hydrolyzes glycerophospholipids to produce free fatty acids and lysophospholipids, directly altering membrane lipid composition and fluidity (Medina et al. 2024). It has been reported that inhibition of the calcium-independent phospholipase A2 (iPLA2) activity of PRDX6 using the specific inhibitor MJ33 significantly reduces mouse sperm motility, viability, and fertility (Moawad et al. 2017). This inhibition also leads to increased lipid peroxidation and enhanced mitochondrial superoxide anion production, indicating that loss of PLA2 activity induces oxidative stress. Furthermore, an sPLA2 inhibitor can block the fertility-promoting effect of PLA2G10 in mice, demonstrating that its catalytic activity is essential for normal fertilization. In this study, the PLA2G10-mediated change in PLA2 activity affects sperm motility through alterations in membrane structure (Abi Nahed et al. 2022). Collectively, these findings support that PLA2G10 is the major PLA2 member mediating the observed changes in total PLA2 activity in sperm. This finding reveals that GABA regulates sperm function through a new mechanism involving membrane remodeling via the phospholipase signaling pathway, rather than relying solely on classical antioxidant protection pathways.

## Conclusion

This study demonstrates that GABA is a critical metabolite for sperm preservation in vitro. Dietary GABA supplementation indirectly improves semen quality and antioxidant capacity in bucks by modulating rumen microbiota composition and enhancing rumen fermentation, as well as by activating the HPT axis. Moreover, under in vitro preservation at 4 °C, adding 1.0 g/L GABA to the semen extender directly acts on sperm by upregulating membrane structure-related proteins and specifically activating PLA2G10-mediated PLA2 activity, thereby remodeling sperm membrane lipid composition and fluidity, which significantly prolongs in vitro sperm preservation time and improves sperm quality. Inhibition of PLA2G10 completely abrogates the protective effects of GABA, confirming that this pathway is the core mechanism underlying its direct action. Taken together, GABA possesses dual functions of in vivo modulation and in vitro protection, making it a promising functional feed additive and semen extender protectant for improving reproductive management in ruminants.

## Supplementary Material

skag294_Supplementary_Data

## Data Availability

The data underlying this article will be shared on reasonable request to the corresponding author.

## Abbreviations

ABC: ATP-binding cassette
ACE: abundance-based coverage estimator
ADG: average daily gain
BSA: bovine serum albumin
BTB: blood-testis barrier
BW: body weight
CASA: computer-assisted sperm analysis
CAT: catalase
CLA: conjugated linoleic acid
CNS: central nervous system
DTT: Dithiothreitol
;ELISA: enzyme-linked immunosorbent assay
FDR: False Discovery Rate
FITC-PNA: fluorescein isothiocyanate-labeled peanut agglutinin
FSH: follicle-stimulating hormone
GAA: guanidinoacetic acid
GABA: gamma-aminobutyric acid
GABARs: GABA receptors
GAD: glutamate decarboxylase
GATs: GABA transporters
GnRH: gonadotropin-releasing hormone
GO: Gene Ontology
GSEA: Gene Set Enrichment Analysis
GSH-Px: glutathione peroxidase
HPG: hypothalamic-pituitary-gonadal
HPT: hypothalamic-pituitary-testicular
IACUC: Institutional Animal Care and Use Committee
iPLA2: calcium-independent phospholipase A2
KEGG: Kyoto Encyclopedia of Genes and Genomes
LH: luteinizing hormone
MDA: malondialdehyde
mTOR: mammalian target of rapamycin
NH₃-N: ammonia nitrogen
OTUs: operational taxonomic units
PBS: phosphate-buffered saline
PCA: principal component analysis
PCoA: principal coordinates analysis
PLA2: phospholipase A2
PLA2G10: group X secreted phospholipase A2
PRDX6: peroxiredoxin 6
ROS: reactive oxygen species
SCFAs: short-chain fatty acids
SDS-PAGE: sodium dodecyl sulfate–polyacrylamide gel electrophoresis
SDT: sodium dodecyl sulfate-Tris
SOD: superoxide dismutase
sPLA2: secreted phospholipase A2
SYBR-14/PI: SYBR-14/propidium iodide
T: testosterone
T-AOC: total antioxidant capacity
TEAB: triethylammonium bicarbonate
UHPLC: ultra-high performance liquid chromatography
VFAs: volatile fatty acids
VIP: Variable Importance in Projection
ω-3 PUFA: omega-3 polyunsaturated fatty acids
