# Supplementary material for: Gamma-aminobutyric acid modulates buck semen quality and sperm preservation duration in vivo and in vitro
Source: J Anim Sci. 2026 Sep 15;104:skag294. doi: 10.1093/jas/skag294 (PMC13600674; doi:10.1093/jas/skag294)
Supplement: Supplementary file 1 [file skag294_supplementary_data.zip › supplementary file.docx]

**supplementary file**

Fig. S1 Effects of GABA on species composition and function of rumen microorganisms at genus level. (A) Microbial species composition analysis (genus level); (B) Microbial community function prediction (tool: Tax4Fun).

Fig. S2 Concentration screening of LY329722 and its effect on sperm antioxidant capacity. (A) LY329722 concentration screening based on sperm motility; (B) Sperm T-AOC; (C), (D) and (E) CAT, GSH-Px and SOD activity; (F) MDA level. Data were expressed as the mean ± SD (n = 3). ^**^ indicated *P* < 0.01.
